# Supplementary figures and images for: Interkinetic nuclear movements promote apical expansion in pseudostratified epithelia at the expense of apicobasal elongation
Source: PLoS Comput Biol. 2019 Dec 23;15(12):e1007171. doi: 10.1371/journal.pcbi.1007171 (PMC6957215; doi:10.1371/journal.pcbi.1007171)

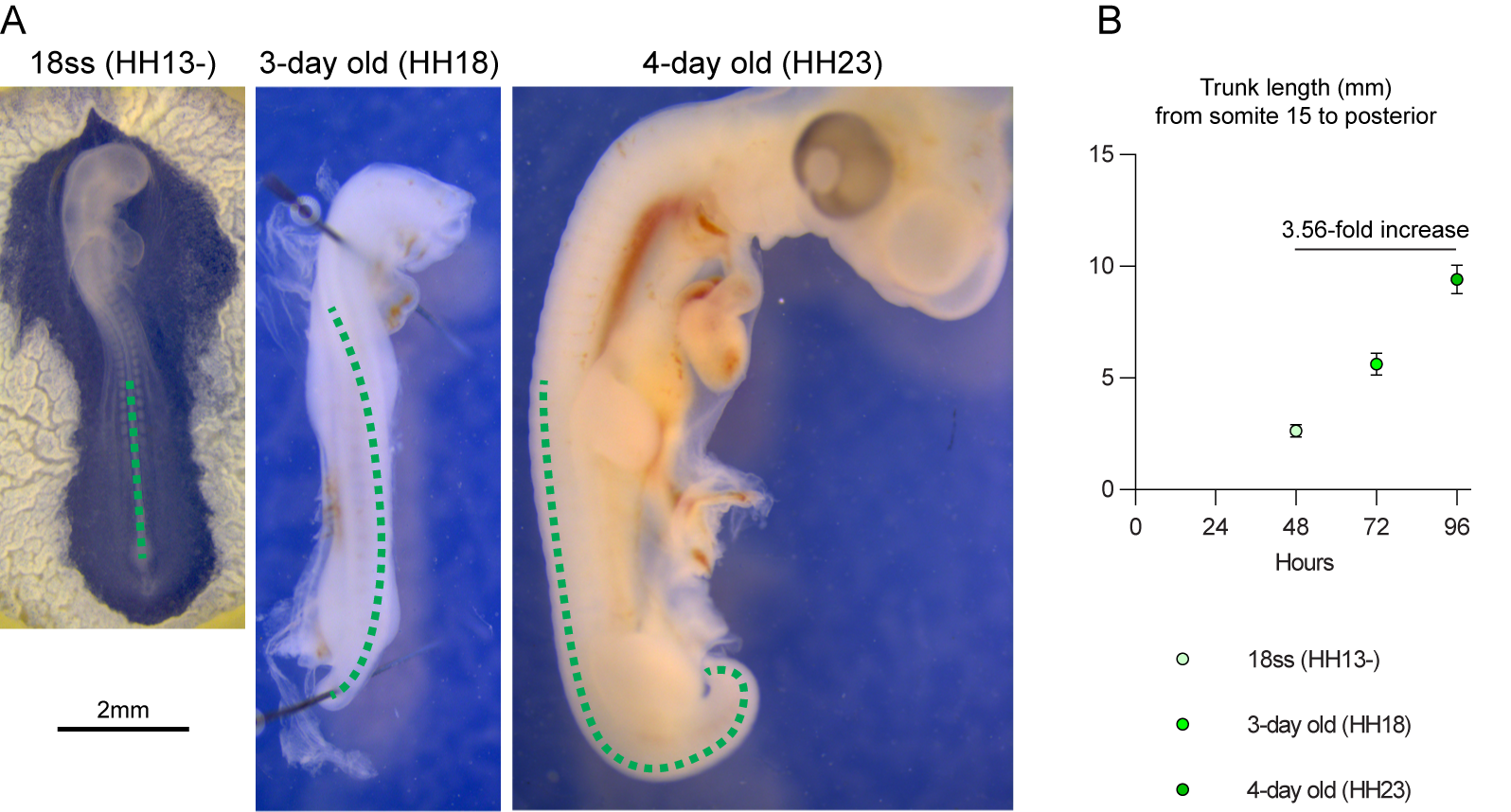

Supplement: S1 Fig — A. Representative images of chicken embryos at stage HH13-, 18 and 23 corresponding to 48, 72 and 96 hours of incubation. The cephalic region of the embryo at stage HH18 removed prior to the picture. The green dotted line indicates the regions that was measured. B, plot of the mean length of the portion indicated in green in panel A. Dots represent the mean, error bars indicate standard deviation. Raw data of all plots provided in the S1 Spreadsheet. (TIF) [file pcbi.1007171.s001.tif]

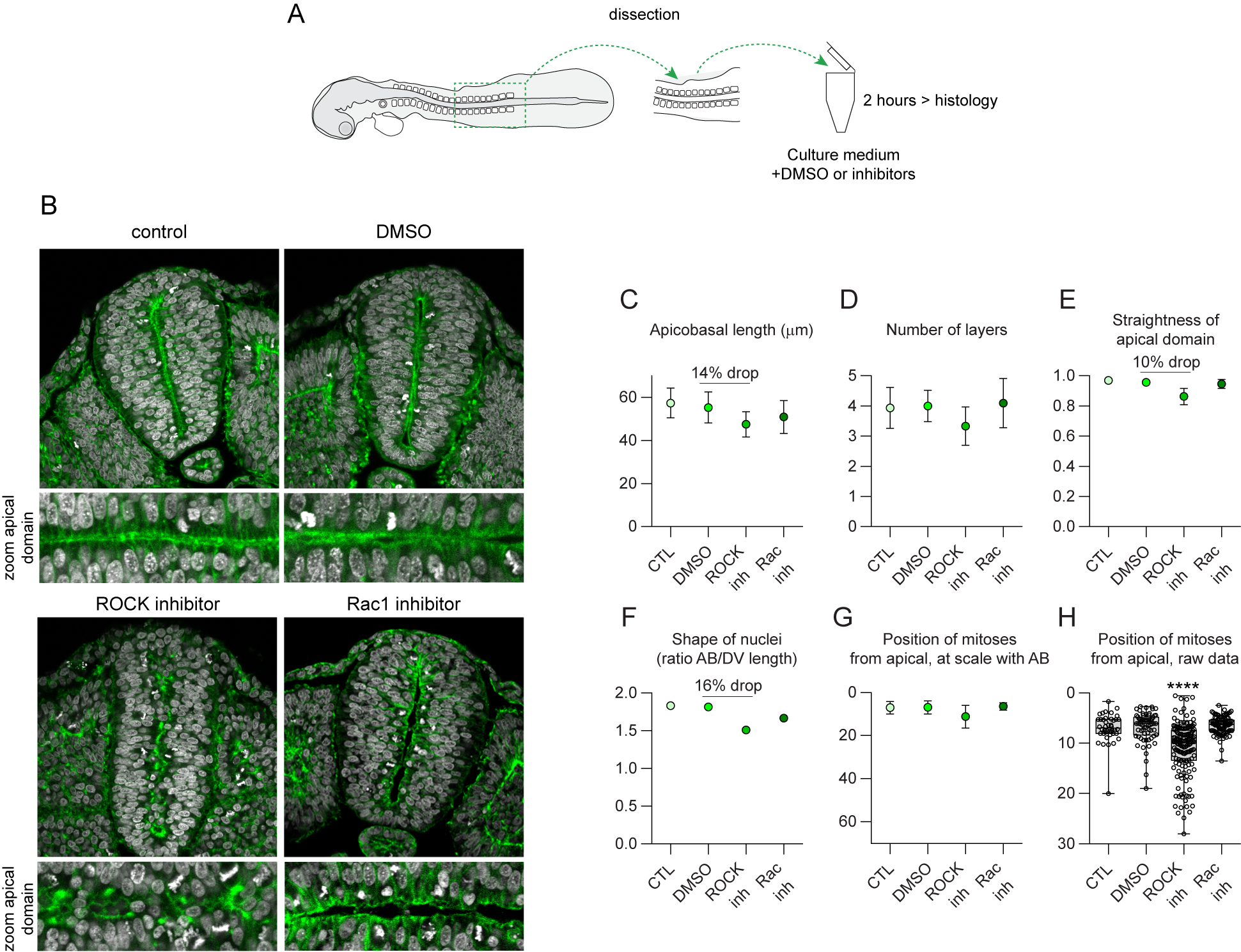

Supplement: S2 Fig — A, Explants of the trunk are incubated in suspension with culture medium, culture medium with DMSO or culture medium with ROCK inhibitor (Y27632, 400μM) or Rac1 inhibitor (NSC27633, 400μM). B, Transversal sections with nuclear (DAPI, grey) and actin staining (Phalloidin, green). C, apicobasal length. D, number of pseudolayers of nuclei. E, straightness of the apical domain. F, shape of nuclei. G, position of mitoses, at scale with the tissue. H, position of mitoses, raw data (embryos/mitoses: ncontrol = 2/42, nDMSO = 2/66, nROCK = 3/153, nRAC1 = 4/98; Kruskal-Wallis followed by multiple comparisons; ****, p<0.0001). Box and whiskers plot: the box extends from the 25th to the 75th percentile; the whiskers show the extent of the whole dataset. The median is plotted as a line inside the box. Raw data of all plots provided in the S1 Spreadsheet. (TIF) [file pcbi.1007171.s002.tif]

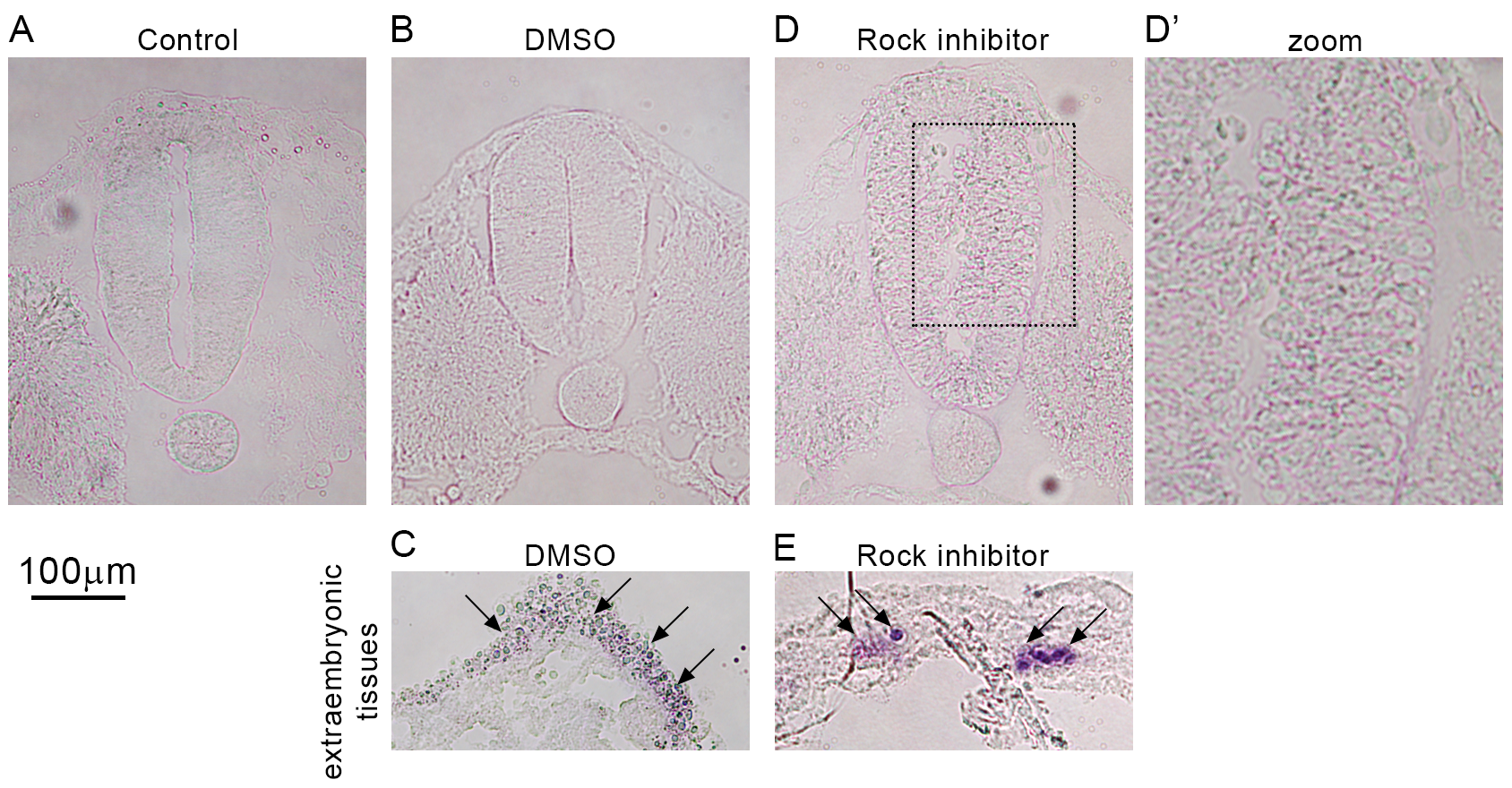

Supplement: S3 Fig — A-E, transversal sections of chick trunk explants after a 2-hour incubation in suspension in culture medium (A, control; n = 4 embryos), supplemented with DMSO (B-C, DMSO; n = 6 embryos) or with the ROCK inhibitor at 400μm (D-E; n = 5 embryos) followed by fixation, TUNEL assays revealed by NBT/BCIP (purple precipitate) and cryosectioning. C and E show portions of the extraembryonic tissues where positive cells for TUNEL were detected in the DMSO and ROCK inhibitor conditions. No TUNEL staining was found in the surface ectoderm, the neural tube, the paraxial mesoderm nor the notochord in either condition. D’, is a zoom of the ROCK inhibitor condition where one can see that tissue deformation caused by the treatment is not accompanied by a massive induction of cell death (no TUNEL-positive cells). All images are at the same scale apart from the zoom in D’ which is magnified twice compared to its cognate low magnification image shown in D. (TIF) [file pcbi.1007171.s003.tif]

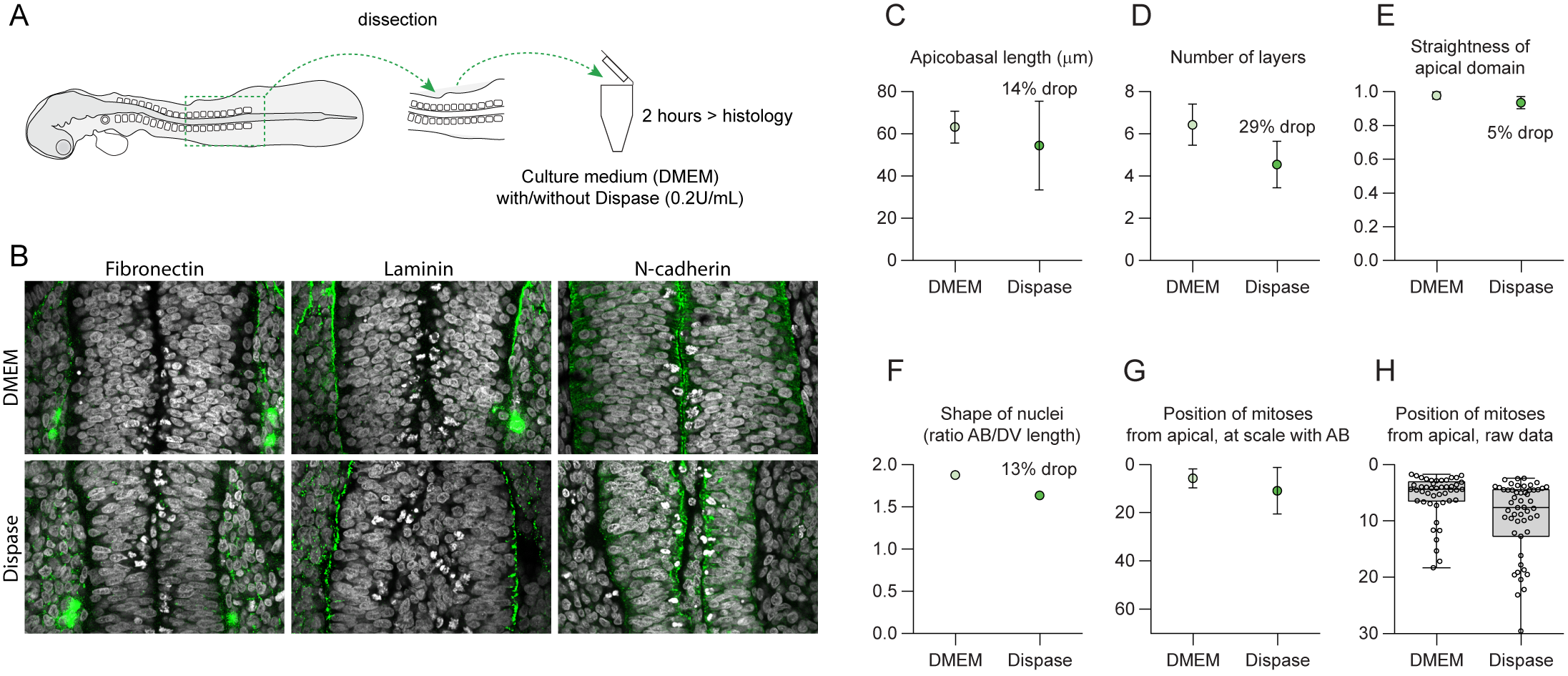

Supplement: S4 Fig — A, Explants of the trunk are incubated in suspension with culture medium (DMEM; n = 6 embryos) or culture medium with Dispase (0.2U/mL; n = 6 embryos). B, Transversal sections with nuclear staining (DAPI, grey) and immunostaining for Fibronectin, laminin or N-cadherin (green). C, apicobasal length. D, number of pseudolayers of nuclei. E, straightness of the apical domain. F, shape of nuclei. G, position of mitoses, at scale with the tissue. H, position of mitoses, raw data (nDMEM = 46, nDispase = 46; **non-parametric t-test p = 0.0017). Box and whiskers plot: the box extends from the 25th to the 75th percentile; the whiskers show the extent of the whole dataset. The median is plotted as a line inside the box. Raw data of all plots provided in the S1 Spreadsheet. (TIF) [file pcbi.1007171.s004.tif]

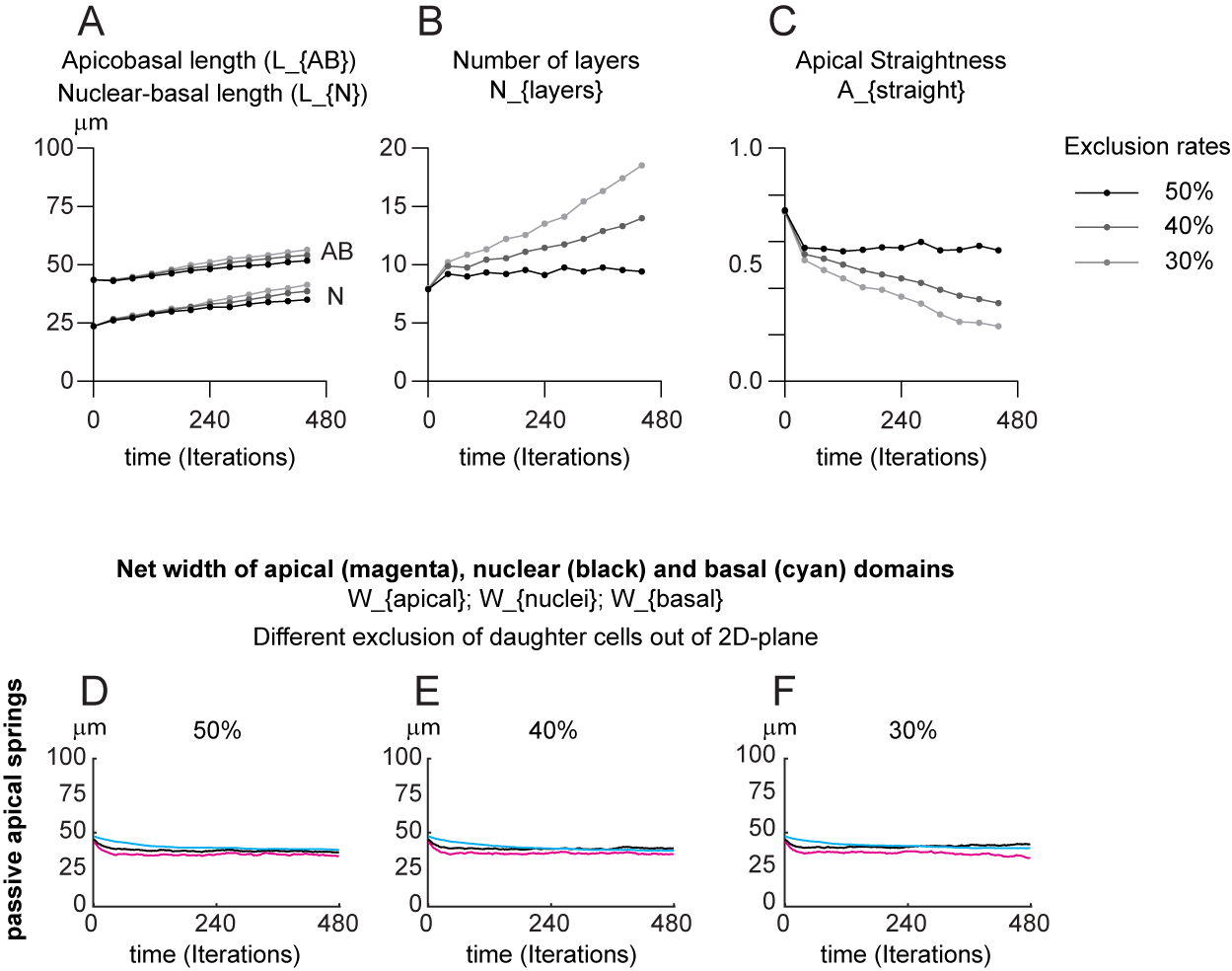

Supplement: S5 Fig — Simulations with passive apical springs, normal INM with different rates of exclusion of daughter cells as in Fig 5 and lateral walls. A, apicobasal length of the PSE (AB) and mean nuclear position along the AB axis (N) over time expressed in micrometers (see S5 Movie). B, Number of pseudolayers of nuclei along the AB axis. C, straightness of apical domain (net distance between the first and last apical point divided by the actual distance between these two points). D-F, net width of apical (magenta), nuclear (black) and basal (cyan) domains of the PSE with 50 (D), 40 (E) and 30% (F) of daughter cells being excluded from the 2D plane. For each domain, the net distance between the first and last point along the lateral axis is computed and its evolution plotted over time. Note that for all conditions the net width of each domain is constant over time confirming that the imposed lateral walls are indeed preventing lateral expansion. Raw data of all plots provided in the S1 Spreadsheet. (TIF) [file pcbi.1007171.s005.tif]

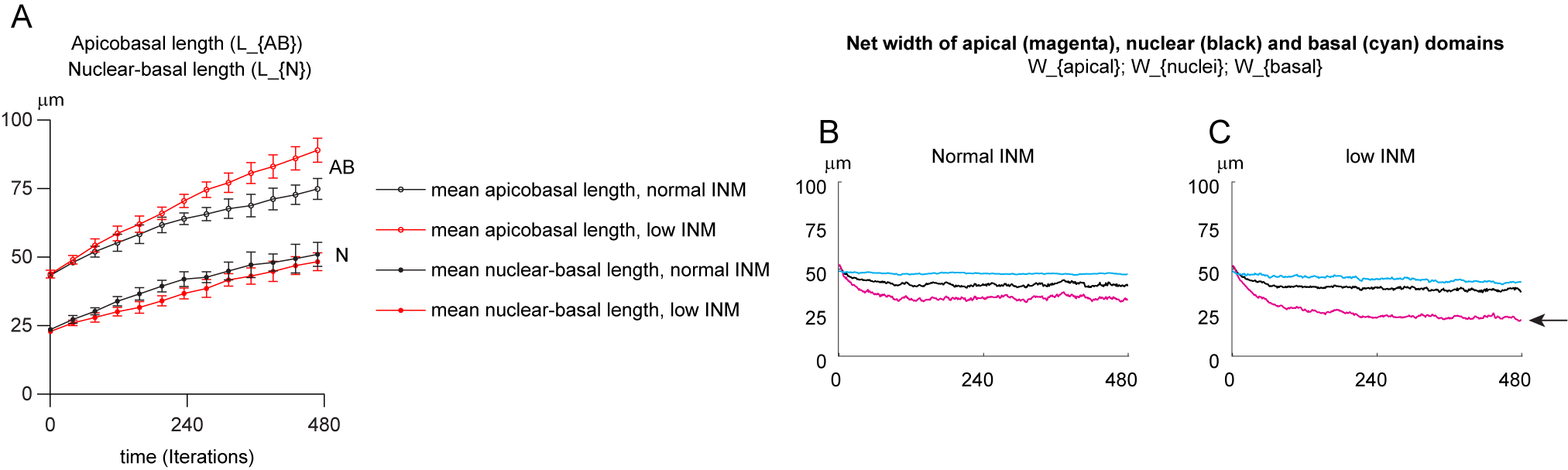

Supplement: S6 Fig — A, Mean apicobasal (AB, open circle) and nuclear-basal (N, closed circles) lengths with normal INM (black) and low INM (red). Note that mean tissue height goes from 75μm to 89μm in absence of INM. In the meantime, mean nuclear height goes from 51μm to 48μm representing a shift from being located within the most apical top third of the tissue with normal INM (51μm /75μm = 68%) to being located midway between the apical and basal domains (48μm/89μm = 54%). B-C, Net width of apical (magenta), nuclear (black) and basal (cyan) domains with normal INM (B) or low INM conditions (C). Note that low INM conditions lead to a shrinkage of the apical domain (arrow). Error bars represent standard deviation. Raw data of all plots provided in the S1 Spreadsheet. (TIF) [file pcbi.1007171.s006.tif]

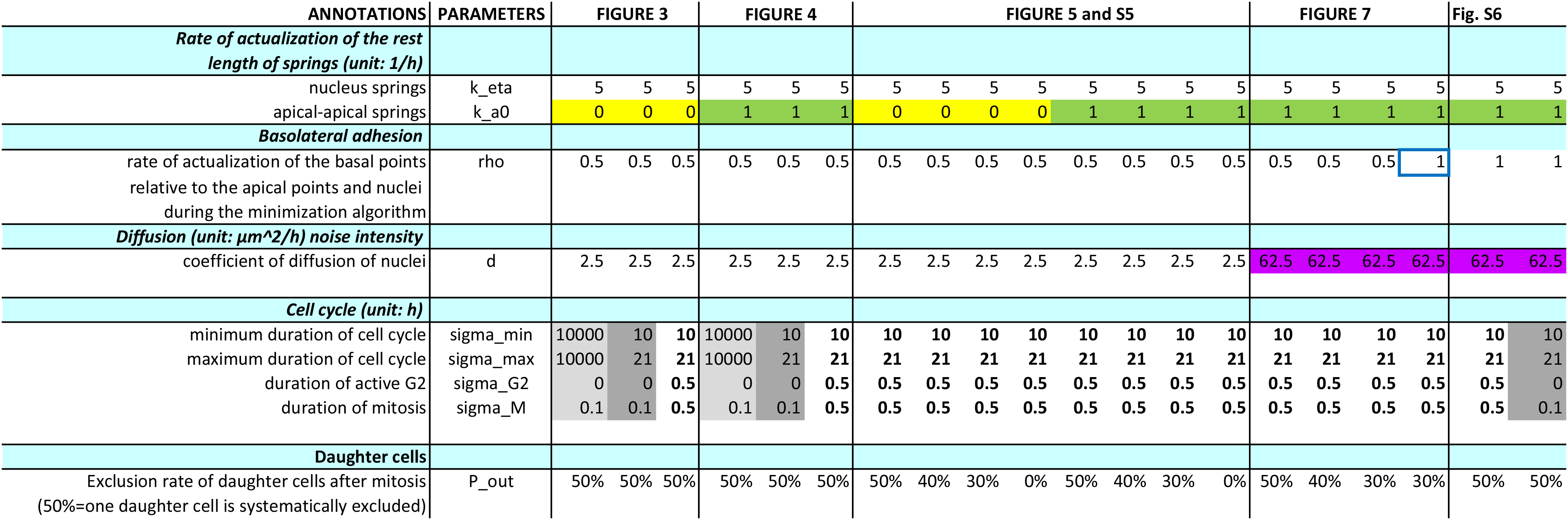

Supplement: S1 Table — Yellow, passive apical-apical springs; Green, contractile apical-apical springs; blue frame, fast update of basal points; magenta, high random nuclear movement (noise); light grey, no proliferation and no INM; dark grey, low INM; bold text, normal INM conditions. The following parameters were common to all simulations: radius of nucleus soft core (Rs = 5μm), radius of nucleus hard core (Rh_S = 1.5μm), radius of nucleus hard core during mitosis (Rh_M = 3.5μm), maximum distance between two consecutive apical points (a0 = 1/6*Rs), maximum distance between two consecutive basal points (b0 = 1/6*Rs). The relative strengths of the various forces was set as follows: stiffness of soft core of the nucleus (alpha_X = 1), stiffness of apical-nucleus spring (alpha_aX = 2), stiffness of nucleus-basal spring (alpha_bX = 2), stiffness of apical-apical spring (alpha_aS = 5), stiffness of apical-apical spring during G2 phase and mitosis (alpha_aM = 10), magnitude of the apical-nucleus-basal alignment force (alpha_ab = 15). (TIF) [file pcbi.1007171.s007.tif]
