# Supplementary material for: Interkinetic nuclear movements promote apical expansion in pseudostratified epithelia at the expense of apicobasal elongation
Source: PLoS Comput Biol. 2019 Dec 23;15(12):e1007171. doi: 10.1371/journal.pcbi.1007171 (PMC6957215; doi:10.1371/journal.pcbi.1007171)
Supplement: S1 Information — (PDF) [file pcbi.1007171.s014.pdf]

Supplementary information

1 Agent-based model of the PSE

1.1 Morphology of the tissue

Let N be the number of cells in the tissue at a given time. A cell i is constituted by the nucleus with center $X_i = (X_i(1), X_i(2)) \in \mathbb{R}^2$, an apical point $a_i = (a_i(1), a_i(2)) \in \mathbb{R}^2$ and a basal point $b_i = (b_i(1), b_i(2)) \in \mathbb{R}^2$. The nucleus is constituted by an inner sphere (the hard core) and an outer sphere (the soft core) with radii $R_i^H > 0$ and $R_i^S > 0$, respectively. The stiffness of the soft core of the nucleus is given by α_i^X. The apical and basal points are connected to the center of the nucleus through springs, representing the cytoplasm. The apical-nucleus spring has rest length $(\eta_i^{aX} + 1)R_i^S$ and stiffness α_i^{aX}. Analogously, the nucleus-basal spring has rest length $(\eta_i^{bX} + 1)R_i^S$ and stiffness α_i^{bX}. Neighbouring apical points j and $j + 1$ are connected to each other by a spring with rest length $2a_{0,j}\bar{R}$, where $\bar{R} = \sum_i R_i^S/N$, and stiffness α_j^a. The apical points and their connections form the apical network. We consider an alignment force between the apical point, nucleus and basal point with magnitude α_i^a, which models apicobasal polarity. The basal points are restricted to the basement membrane, which is represented by a horizontal line, i.e., $b_i(2)$ is kept constant for all i. Neighbouring basal points cannot switch positions nor get too far away from each other (lateral adhesion), i.e., $0 < b_{i+1}(1) - b_i(1) < 2\bar{R}b_0,\ i = 1, ..., N-1$, where $b_0 > 0$. Basal adhesion is controlled by a parameter ρ that influences the displacement of the basal points during the minimization algorithm as described in Section 2.1. To assess the effect of restricting lateral expansion of the tissue we will sometimes consider two lateral rigid walls perpendicular to the basement membrane and passing through $(x_L, 0)\bar{R}$ and $(x_R, 0)\bar{R}$, with $x_L,\ x_R \in \mathbb{R}$ appropriately chosen. In this case the center of nuclei will be restricted to $x_L\bar{R} + R_i^H \leq X_i(1) \leq x_R\bar{R} - R_i^H,\ i = 1, ..., N$.

1.2 Tissue at mechanical equilibrium: minimization problem

We assume that the tissue is always trying to reach a state of minimum energy, corresponding to a mechanical equilibrium. This equilibrium is obtained at each time-step as a solution to a minimization problem for the centers of nuclei, apical points and basal points. All the other parameters are constants that are determined by the dynamics of the system, as explained in the next Section. We minimize a potential corresponding to the sum of the energy associated to the springs, the alignment force and the soft core of the nucleus. The minimization is subject to non-overlapping constraints on the nuclei inner-cores and non-switching and adhesion constraints on the basal points. Let $\mathbf{X} = (X_1, ..., X_N)$, $\mathbf{a} = (a_1, ..., a_N)$

and $\mathbf{b} = (b_1, ..., b_N)$ be the positions of the centers of nuclei, apical points and basal points, respectively. Consider the parameters defined in the previous Section. We define the potential $W : (\mathbb{R}^{2N})^3 \to \mathbb{R}$ by

$$W = W_1 + W_2 + W_3 + W_4 + W_5$$

with

$$\begin{aligned}
W_1(\mathbf{X}, \mathbf{a}, \mathbf{b}) &= \sum_{i=1}^{N} \alpha_i^{aX} \left| \frac{|a_i - X_i|}{R_i^S(1+\eta_i^{bX})} - 1 \right|^2, \\
W_2(\mathbf{X}, \mathbf{a}, \mathbf{b}) &= \sum_{i=1}^{N} \alpha_i^{bX} \left| \frac{|b_i - X_i|}{R_i^S(1+\eta_i^{bX})} - 1 \right|^2, \\
W_3(\mathbf{X}, \mathbf{a}, \mathbf{b}) &= \sum_{i=1}^{N-1} \alpha_i^{a} \left| \frac{|a_{i+1} - a_i|}{2\bar{R}} - a_0 \right|^2, \\
W_4(\mathbf{X}, \mathbf{a}, \mathbf{b}) &= \sum_{i=1}^{N} \alpha_i^{ab} \left(\frac{(a_i - X_i) \cdot (b_i - X_i)}{|a_i - X_i||b_i - X_i|} + 1 \right)^2 \\
\text{and } W_5(\mathbf{X}, \mathbf{a}, \mathbf{b}) &= \sum_{i,j=1}^{N} \alpha_{ij}^{X} \mathbb{1}_{\{|X_i - X_j| < R_i^S + R_j^S\}}(\mathbf{X}) \left| \frac{|X_i - X_j|}{R_i^S + R_j^S} - 1 \right|^2,
\end{aligned}$$

where $|\cdot|$ denotes the Euclidean norm and $\mathbb{1}_A$ denotes the indicator function of the set A, i.e., the function that takes the value 1 on A and the value 0 on its complement set. The potentials W_1, W_2 and W_3 represent the energy associated to the apical-nucleus, nucleus-basal and apical-apical springs, respectively, W_4 represents the energy associated to the alignment forces and W_5 to the soft core of the nuclei. Note that $W \geq 0$ and $W = 0$ if and only if all springs are at their rest position, the apical point, nucleus and basal point of each cell are aligned and the nucleus soft cores are not overlapping with each other. For each $k, \ell = 1, ..., N,\ k < \ell$ define the function associated to the non-overlapping constraint between nuclei k and ℓ:

$$\phi_{k\ell}(\mathbf{X}) = \frac{(R_k^H + R_\ell^H)^2 - |X_k - X_\ell|^2}{\bar{R}^2}.$$

For each $i = 1, ..., N-1$ define the functions associated to the non-switching and lateral adhesion constraints between basal points i and $i+1$:

$$\psi_i(\mathbf{b}) = \frac{b_i(1) - b_{i+1}(1)}{\bar{R}} \text{ and } \Omega_i(\mathbf{b}) = \frac{b_{i+1}(1) - b_i(1)}{\bar{R}} - 2b_0, \quad \text{respectively.}$$

The minimization problem is formulated as follows: find $(\bar{\mathbf{X}}, \bar{\mathbf{a}}, \bar{\mathbf{b}})$ such that $b_i(2) = 0$ for all i and

$$(\bar{\mathbf{X}}, \bar{\mathbf{a}}, \bar{\mathbf{b}}) \in \underset{\substack{\psi_i(\mathbf{b}),\ \Omega_i(\mathbf{b}) \leq 0,\ \forall i \\ \phi_{k\ell}(\mathbf{X}) \leq 0,\ \forall k,\ell,\ k<\ell}}{\operatorname{argmin}} W(\mathbf{X}, \mathbf{a}, \mathbf{b}) \tag{1.1}$$

Due to the non-convexity of $\phi_{k\ell}$, a solution to this minimization problem is not unique. Therefore a configuration $(\bar{\mathbf{X}}, \bar{\mathbf{a}}, \bar{\mathbf{b}})$ obtained at each time-step corresponds to a local minimizer. This is consistent with the dynamics of the tissue in vivo. Indeed, the movement of

cells is continuous, i.e., cells are not observed to jump large distances during a small time interval. Consequently, each cell has to search locally for a state of lower energy. Such local search yields a local minimizer of the potential energy.

In the presence of two lateral walls we impose $2N$ extra constraints, $\chi_i^R(\mathbf{X}) \leq 0$, $\chi_i^L(\mathbf{X}) \leq 0$, $i = 1, ..., N$, with

$$\chi_i^R(\mathbf{X}) = \frac{X_i(1) + R_i^H}{\bar{R}} - x_R \quad \text{and} \quad \chi_i^L(\mathbf{X}) = x_L + \frac{R_i^H - X_i(1)}{\bar{R}},$$

which ensures that all nuclei remain in between the two walls.

1.3 Dynamics driven by noise, cell division and changes in cell characteristics

In this Section we describe the time-dependent model for the dynamics and growth of the tissue during the time interval $[0, T]$, where $T > 0$. The dynamics of the tissue is driven by noise, cell division and by the search for a state of minimal energy as described in Section 1.2. Next we present the dynamics of the springs, noise and walls, followed by the dynamics of the cell cycle.

In order to accommodate the increasing number of nuclei, the tissue will grow in all directions. Consequently, this will lead apical-nucleus, nucleus-basal and apical-apical springs to stretch. We assume that cells release tension by letting the rest lengths progressively converge to the current cell configuration. This models the dynamics of actomyosin and microtubules inside a cell. Let the configuration of cell i at some time t, $(X_i(t), a_i(t), b_i(t))$, be given. The preferred apical-nucleus and nucleus-basal rest lengths at time t are respectively given by

$$\eta_i^{aX*}(t) = \frac{|X_i(t) - a_i(t)|}{R_i^S} - 1 \qquad \text{and} \qquad \eta_i^{bX*}(t) = \frac{|X_i(t) - b_i(t)|}{R_i^S} - 1. \tag{1.2}$$

The dynamics of the rest lengths η_i^{aX} and η_i^{bX} are governed by the ODEs

$$\dot{\eta}_i^{aX}(t) = \begin{cases} 0, & \text{if } \eta_i^{aX}(t) = 0 \text{ and } \eta_i^{aX*}(t) < \eta_i^{aX}(t) \\ k_\eta(\eta_i^{aX*}(t) - \eta_i^{aX}(t)), & \text{otherwise} \end{cases}, \quad t \geq 0 \tag{1.3}$$

and

$$\dot{\eta}_i^{bX}(t) = \begin{cases} 0, & \text{if } \eta_i^{bX}(t) = 0 \text{ and } \eta_i^{bX*}(t) < \eta_i^{bX}(t) \\ k_\eta(\eta_i^{bX*}(t) - \eta_i^{bX}(t)), & \text{otherwise} \end{cases}, \quad t \geq 0 \tag{1.4}$$

where $\dot{\eta}$ represents the time derivative of η and $k_\eta > 0$ is the speed of actualization of the rest lengths. Note that even if the preferred rest lengths $\eta_i^{aX*}(t)$ and $\eta_i^{bX*}(t)$ may be negative at some time t, the rest lengths $\eta_i^{aX}(t)$ and $\eta_i^{bX}(t)$ remain non-negative for all times. Apical-apical springs may be passive or contractile, if the rest lengths are constant over time or if they decrease towards zero, respectively. The dynamics of the rest length $a_{0,i}$ follows the ODE

$$\dot{a}_{0,i}(t) = -k_{a_0} a_{0,i}(t), \qquad t \geq 0 \tag{1.5}$$

where k_{a_0} is the speed of actualization of the rest length. We set $k_{a_0} = 0$ in the case of passive springs and $k_{a_0} > 0$ in the case of contractile springs. Nuclei diffuse with coefficient of diffusion d. Specifically, for each cell i, the vector X_i follows the stochastic differential equation

$$dX_i(t) = \sqrt{2d}\, dW(t), \tag{1.6}$$

where $W(t)$ is the two-dimensional standard Wiener process. This models the random displacements of nuclei, i.e., noise, observed in vivo. The diffusion coefficient d gives a measure of the noise intensity. The positions of the walls $x_L(t)$ and $x_R(t)$ are determined by the ODEs

$$\dot{x}_L(t) = \begin{cases} 0, & \text{if } k_L(t) \leq 1 \\ -k_L(t), & \text{otherwise} \end{cases} \quad \text{and} \quad \dot{x}_R(t) = \begin{cases} 0, & \text{if } k_R(t) \leq 1 \\ k_R(t), & \text{otherwise} \end{cases}, \quad t \geq 0, \tag{1.7}$$

where $k_L(t) \geq 0$ and $k_R(t) \geq 0$ represent a measure of the intensity of the force that the cells exert on the walls. The explicit formulas for k_L and k_R will be specified in the next Section.

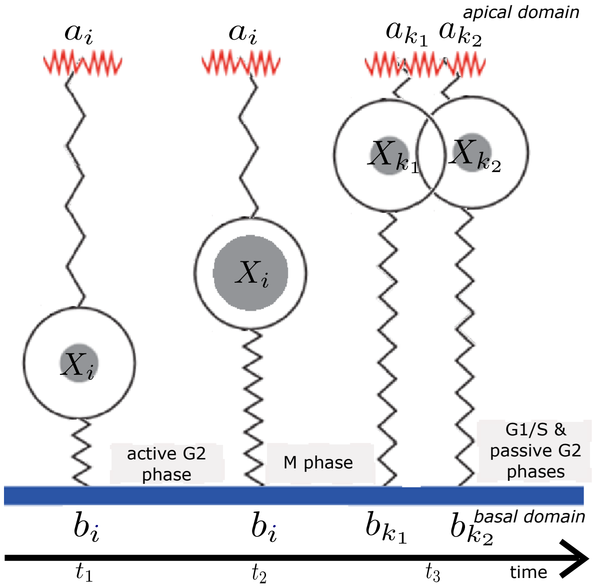

Figure 1: Sketch of the changes occurring to a cell prior to division. During active G2 phase and M phase, the preferred rest length of nucleus-apical and basal-nucleus springs are set to $\eta_i^{aX*} = 0$ and $\eta_i^{bX*} = |a_i - b_i|/R_i^S - 2$, respectively, which drives the nucleus apicalwards. At the beginning of M-phase, the radius of the hard core of the nucleus (grey circle) increases. At the end of M-phase, division occurs: cell i is substituted by two cells k_1 and k_2 and the springs and nuclei are reset to the values prior to G2 phase. During G1/S and passive G2 phases the rest lengths of nucleus springs evolve to minimize local tensions.

We consider the cell cycle divided into three stages: G1/S, active G2 and mitosis (M). We assume that a cell has an internal clock that determines the position of the cell in the cell cycle. We denote by σ_{G2} and σ_M the duration of G2 and M phases, respectively. Moreover, for each cell i, τ_i denotes the birth time and $\sigma_i \in [\sigma_{\min}, \sigma_{\max}]$, with $\sigma_{\max} > \sigma_{\min} > 0$, denotes the duration of the cell cycle. During the first stage G1/S, a cell i moves passively driven by

noise and by the local environment. The stiffness of the apical-apical spring is $\alpha_i^a = \alpha^{a,S}$, the radius of the hard core is $R_i^H = R^{HS}$ and the preferred rest lengths of the nucleus springs are given by (1.2). During active G2 stage, the characteristics of the springs change so that the nucleus is going to actively try to approach the apical side of the tissue: the apical-apical spring gets stiffer ($\alpha_i^a = \alpha^{a,G2}$) and a pushing and a pulling forces are activated to drive the nucleus apicalwards (the rest lengths of the nucleus springs are set to $\eta_i^{aX*} = 0$ and $\eta_i^{bX*} = |a_i - b_i|/R_i^S - 2$). During M stage, the changes implemented during G2 are kept and the hard core of the nucleus gets larger ($R_i^H = R^{HM}$). A cell will divide as soon as it reaches the end of the cell cycle, even if the nucleus has not been able to reach the apical side. The division plan is parallel to the basement membrane. One daughter cell is excluded from the 2D domain in consideration with probability P_{out}. Cell division is performed by substituting the mother cell i by one or two daughter cells, k_1 or k_1 and k_2, according to the following rules (see Figure 1):

(a) if no daughter cell has been excluded from the domain, then N is incremented by 1, the centeres of the nuclei of the daughter cells are located at $X_{k_1} = X_i + p_i$ and $X_{k_2} = X_i - p_i$, with $p_i = (0.05R_i^S, 0)$ and the apical and basal points are located at $a_i \pm p_i$ and $b_i \pm p_i$, respectively. Otherwise, if one cell is excluded, then N remains constant and the daughter cell gets the same position as the mother cell.

(b) the radius of the hard core of the nucleus and the stiffness of the apical-apical springs are restored to the values prior to G2 phase: $R_{k_1}^H = R^{HS}$ and $\alpha_{k_1}^a = \alpha^{a,S}$, respectively, ($R_{k_2}^H = R^{HS}$, $\alpha_{k_2}^a = \alpha^{a,S}$).

(c) the birth time is defined by $\tau_{k_1} = t_n$, ($\tau_{k_2} = t_n$) and the lengths of the cycle of the cell(s) are generated from the uniform distribution $\sigma_{k_1}, (\sigma_{k_2}) \in \mathcal{U}([\sigma_{\min}, \sigma_{\max}])$,

(d) all the remaining cell parameters associated to the rest lengths of the springs and magnitude of the forces inherit the same values as the mother cell.

We distinguish between model and cell parameters. Model parameters are non-negative constants and they are listed in Table 1, Section 3. Cell parameters are time-dependent vectors with dimension N or $2N$ denoted in bold, namely, cell positions, $\mathbf{X}, \mathbf{a}, \mathbf{b}$, nucleus radii, $\mathbf{R}^S, \mathbf{R}^H$, strength of forces, $\boldsymbol{\alpha}^{aX}, \boldsymbol{\alpha}^{bX}, \boldsymbol{\alpha}^{a}, \boldsymbol{\alpha}^{ab}, \boldsymbol{\alpha}^{X}$, rest lengths of springs, $\boldsymbol{\eta}^{aX}, \boldsymbol{\eta}^{bX}, \mathbf{a}_0$, duration of cell cycle $\boldsymbol{\sigma}$ and birth time $\boldsymbol{\tau}$. Note that in the present model, the second coordinate of the basal points $b_i(2)$, the radius of the soft core $R_i^S = R^S$ and the stiffness $\alpha_i^{aX} = \alpha^{aX}$, $\alpha_i^{bX} = \alpha^{bX}$, $\alpha_i^X = \alpha^X$ and $\alpha_i^{ab} = \alpha^{ab}$ remain constant for all cells i and all times t.

2 Algorithms and numerical parameters

2.1 Minimization algorithm

Here we describe the damped Arrow-Hurwicz algorithm (DAHA) to approximate a solution to the minimization problem (1.1) described in Section 1.2. The DAHA has been developed in [1] from the original Arrow-Hurwicz algorithm to tackle minimization problems with non-convex

constraints, such as the non-overlapping constraints $\phi_{k\ell} \leq 0$. We consider the Lagrangian $\mathcal{L} : (\mathbb{R}^{2N})^3 \times \mathbb{R}_+^{N(N-1)/2} \times (\mathbb{R}_+^{N-1})^2 \to \mathbb{R}$ defined by

$$\mathcal{L}(\mathbf{X}, \mathbf{a}, \mathbf{b}, \boldsymbol{\lambda}, \boldsymbol{\mu}, \boldsymbol{\xi}) = W(\mathbf{X}, \mathbf{a}, \mathbf{b}) + \sum_{k,\ell=1,\dots,N,\ k<\ell} \lambda_{k\ell}\phi_{k\ell}(\mathbf{X}) + \sum_{i=1}^{N-1} \mu_i \psi_i(\mathbf{b}) + \sum_{i=1}^{N-1} \xi_i \Omega_i(\mathbf{b})$$

where $\boldsymbol{\lambda} = \{\lambda_{k\ell}\}_{k,\ell=1,\dots,N,\ k<\ell}$, $\boldsymbol{\mu} = \{\mu_i\}_{i=1,\dots,N-1}$ and $\boldsymbol{\xi} = \{\xi_i\}_{i=1,\dots,N-1}$ are the Lagrange multipliers associated to the constraints. Due to the necessary conditions for optimality [1, 2], a solution to the minimization problem is a steady-state of the second-order damped Arrow-Hurwicz ODE system,

$$\begin{cases} \ddot{X}_i & = -c_1\dot{X}_i - (c_2)^2\nabla_{X_i}\mathcal{L} - (c_3)^2 \displaystyle\sum_{k,\ell=1,\dots,N,\ k<\ell} \lambda_{k\ell}\phi_{k\ell}\nabla_{X_i}\phi_{k\ell} \\ \ddot{a}_i & = -c_1\dot{a}_i - (c_2)^2\nabla_{a_i}\mathcal{L} \\ \ddot{b}_i & = -c_1\dot{b}_i - \rho^2(c_2)^2\nabla_{b_i}\mathcal{L} - (c_3)^2 \displaystyle\sum_{k=1}^{N-1} \left[\mu_k\psi_k\nabla_{b_i}\psi_k + \xi_k\Omega_k\nabla_{b_i}\Omega_k\right], \\ & \qquad\qquad\qquad\qquad i = 1, \dots, N \\ \dot{\lambda}_{k\ell} & = \begin{cases} 0, & \text{if } \lambda_{k\ell} = 0 \text{ and } \phi_{k\ell} < 0 \\ \beta\phi_{k\ell}, & \text{otherwise} \end{cases}, \quad k, \ell = 1, \dots, N,\ k < \ell \\ \dot{\mu}_k & = \begin{cases} 0, & \text{if } \mu_k = 0 \text{ and } \psi_k < 0 \\ \beta\psi_k, & \text{otherwise} \end{cases}, \quad k = 1, \dots, N-1 \\ \dot{\xi}_k & = \begin{cases} 0, & \text{if } \xi_k = 0 \text{ and } \Omega_k < 0 \\ \beta\Omega_k, & \text{otherwise} \end{cases}, \quad k = 1, \dots, N-1 \end{cases} \tag{2.8}$$

where c_1, c_2, c_3 and β are positive constants and $\rho \in [0, 1]$. Given an initial condition $(\hat{\mathbf{X}}, \hat{\mathbf{a}}, \hat{\mathbf{b}}, \hat{\boldsymbol{\lambda}}, \hat{\boldsymbol{\mu}}, \hat{\boldsymbol{\xi}})$, with $(\hat{\boldsymbol{\lambda}}, \hat{\boldsymbol{\mu}}, \hat{\boldsymbol{\xi}}) = 0$, and a small step size $\delta > 0$, the DAHA is obtained by discretizing explicitly the equations in X_i, a_i, b_i and implicitly the equations in $\lambda_{k\ell}$, μ_k, ξ_k. The numerical parameters are given by $(\tilde{c}_1, \tilde{c}_2, \tilde{c}_3, \tilde{\beta}) = (\delta c_1,\ \delta c_2,\ \delta c_3, \delta\beta)$ and the tolerance associated to the stopping criterion is denoted by ε. The values used in the simulations are $\tilde{c}_1 = 2$, $\tilde{c}_2 = 0.01\bar{R}$, $\tilde{c}_3 = 0.1\bar{R}$, $\tilde{\beta} = 0.25$ and $\varepsilon = 10^{-6}$, where $\bar{R}$ is the average radius of the nucleus soft core. The parameter ρ in the equation for b_i represents the rate of actualization of basal points relatively to the rate of actualization of apical points and nuclei during the minimization algorithm. By choosing ρ within $[0, 1)$, the movement of the basal points is slowed down. Since the solution to the minimization problem is not unique (see Section 1.2), a local minimizer $(\bar{\mathbf{X}}, \bar{\mathbf{a}}, \bar{\mathbf{b}})$ will then be selected in which the displacement of the basal points with respect to their initial positions, $|\bar{\mathbf{b}} - \hat{\mathbf{b}}|$, is small compared to the displacement of apical points $|\bar{\mathbf{a}} - \hat{\mathbf{a}}|$ and nuclei $|\bar{\mathbf{X}} - \hat{\mathbf{X}}|$. Consequently, the relative movement of basal points during the dynamics of the tissue is slowed down, which allows us to model basal adhesion. In the presence of lateral walls, we consider additional Lagrange multipliers ν_i^R, ν_i^L, associated to the constraints $\chi_i^R \leq 0$, $\chi_i^L \leq 0$, $i = 1, \dots, N$. These multipliers satisfy differential equations analogous to the remaining multipliers. Moreover, the Lagrangian $\mathcal{L}$ gains an additional term $\sum_{i=1}^{N}[\nu_i^R\chi_i^R(\mathbf{X}) + \nu_i^L\chi_i^L(\mathbf{X})]$, which yields extra terms in the equations for $\mathbf{X}$ in (2.8).

To reduce computational time, we subdivided the domain in squared boxes with side $4\bar{R}$ and searched for overlapping nuclei within the same box or in neighbouring boxes, which allowed to reduce the order of magnitude of the number of equations in $\lambda_{k\ell}$ from N^2 to N.

2.2 Time-stepping scheme

In this Section we present the time-stepping algorithm used to simulate the dynamics described in Section 1.3. Let Δt be the time-step, which in practice will be equal to the time interval between two frames in the videos obtained in the lab, i.e., $\Delta t = 0.1$ hours. Consider the initial time $t_0 = 0$ and the final time T. Let the model parameters be given (see end of Section 1.3). Let the initial number of cells N_0, the values for the initial cell positions $\mathbf{X}_0, \mathbf{a}_0, \mathbf{b}_0$ and rest lengths $\boldsymbol{\eta}_0^{aX}, \boldsymbol{\eta}_0^{bX}, \mathbf{a}_{00}$, the birth times $\boldsymbol{\tau}$ and the durations of cell cycle $\boldsymbol{\sigma}$ be given. Let the remaining cell parameters be initialized according to the cell stage. Given the cell parameters at time t_{n-1}, we obtain the cell parameters at time $t_n = n\Delta t$ by performing the following steps:

1. update of the rest length of nucleus springs: for each cell i we obtain $\eta_{i,n}^{aX}$ and $\eta_{i,n}^{bX}$ through an explicit time-discretization of equations (1.3)-(1.4) with η_i^{aX*} and η_i^{bX*} given by (1.2) if cell i is in G1/S phase and with $\eta_i^{aX*} = 0$ and $\eta_i^{bX*} = |a_{i,n-1} - b_{i,n-1}|/R_i^S - 2$ if cell i is in G2 or M phases.

2. update of the rest length of apical-apical springs: for each cell i, we obtain $a_{0,i,n}$ through an explicit time-discretization of equation (1.5).

3. update of the stiffness of the apical-apical springs of cells k that are going to enter in G2 phase: $\alpha_{k-1}^a = \alpha^{a,G2}$ and $\alpha_k^a = \alpha^{a,G2}$.

4. update of the nucleus rigidity of cells k that are going to enter in M phase: $R_k^H = R^{HM}$.

5. division of cells k that have reached the end of the cell cycle by (i) choosing with probability P_{out} if one of its daughter cells will be excluded from the system and (ii) performing the division by substituting the mother cell by the daughter(s) cell(s) k_1 (and k_2) according to rules a)-d) presented in Section 1.3: obtain $(\hat{\hat{\mathbf{X}}}_n, \hat{\mathbf{a}}_n, \hat{\mathbf{b}}_n)$, update N and the daughter cell(s) parameters.

6. computation of $\hat{\mathbf{X}}_n$ by adding to $\hat{\hat{\mathbf{X}}}_n$ Gaussian white noise with mean 0 and standard deviation $\sqrt{2d\Delta t}$, which corresponds to a time-discretization of equation (1.6) given by: $\hat{\mathbf{X}}_n = \hat{\hat{\mathbf{X}}}_n + \sqrt{2d\Delta t}u$, where u is generated from the $2N$-dimensional multivariate standard Gaussian distribution.

7. computation of an admissible configuration $(\mathbf{X}_n, \mathbf{a}_n, \mathbf{b}_n)$ by readjusting the cell positions $(\hat{\mathbf{X}}_n,\ \hat{\mathbf{a}}_n, \hat{\mathbf{b}}_n)$. In other words, we obtain a local solution, $(\mathbf{X}_n, \mathbf{a}_n, \mathbf{b}_n)$, of (1.1) by numerically solving the ODE system (2.8) with initial condition $(\hat{\mathbf{X}}_n, \hat{\mathbf{a}}_n, \hat{\mathbf{b}}_n)$.

8. if $t_n \geq T$, then the cycle stops.

If the walls are present, their positions $x_{L,n}$ and $x_{R,n}$ are computed between steps 6 and 7 using an explicit time-discretization of (1.7) with $k_{L,n} = \sum_{i=1}^{N} \bar{\nu}_{i,n-1}^{L}$ and $k_{R,n} = \sum_{i=1}^{N} \bar{\nu}_{i,n-1}^{R}$, where $\bar{\nu}_{i,n-1}^{L}$, $\bar{\nu}_{i,n-1}^{R}$, $i = 1, ..., N$, are the Lagrange multipliers obtained by the minimization algorithm (step 7) in the previous time-step $n - 1$.

3 Model parameters, initial and stopping conditions

The ranges for the model parameters were obtained from the literature or from in vivo observations. They are presented in Table 1 together with the values used in the paper. The initial and stopping conditions are presented in Table 2. We denote the space unit by μm (micrometer), the time unit by h (hour) and the unit of strength of a force by α (strength of the soft core of the nucleus). The values for the parameters associated to the cell cycle and exclusion rate of daughter cells are discussed in the main text. For convenience we recall them here: the minimum and maximum duration of cell cycle are $(\sigma_{\min}, \sigma_{\max}) = (10, 21)h$, the duration of G2 and M phases are $(\sigma_{G2}, \sigma_M) = (0.5, 0.5)h$, and the exclusion rate of one daughter cell per division is $P_{\text{out}} \in [0, 100]\%$.

3.1 Nucleus radii

We choose the radius of the soft core of the nuclei R_i^S, $i = 1, ..., N$, such that R_i^S is constant over i and the average value is equal to the average radius of the nucleus, i.e., $\bar{R} = 5\mu m$, therefore $R_i^S = R^S = 5\mu m$, for all $i = 1, ..., N$. Measurements presented in Figure 2I in the main text indicate that the shape of the nucleus is close to an ellipsoid with axis a, b, c, where $a = b < c$ and $c \approx 2a$. We consider R^S equal to the larger axis and R^{HS} is chosen from an interval around the value of the smaller axis, namely, $R^S = 2a$ and $R^{HS} \in [a - R^S/5, a + R^S/5]$, yielding, $R^{HS} \in [0.3, 0.7]R^S$. During mitosis the nucleus swells, therefore the value of the radius of the hard core should be larger than outside mitosis, i.e., $R^{HM} \in [R^{HS}, R^S]$.

3.2 Relative strength of forces

In the chick neuroepithelium cell-cell adhesions are concentrated at the apical pole of each cell and the apical surface remains flat. In addition, cells keep a straight shape, aligned with the apicobasal axis. Therefore, we assume the strength of apical-apical springs and alignment force to be significantly larger than the strength of nucleus soft core and nucleus springs. So we fix $\alpha^X = 1\alpha$ and choose the relative strengths α^{aX}, α^{bX} of order α and $\alpha^{a,S}, \alpha^{a,M}$ and α^{ab} of order 10α. In addition, due to known swelling and stiffening of cells during division, G2-phase and M-phase, the strength of apical-apical spring should be larger than outside these stages, therefore $\alpha^{a,M} > \alpha^{a,S}$.

3.3 Rate of actualization of the rest length of springs

During the active G2 phase, the nucleus migrates rapidly to the apical side. This implies that the time of actualization of the rest lengths of the nucleus springs, $1/k_\eta$, has to be smaller or equal than the duration of G2 phase, $\sigma_{G2} = 0.5$ hours, so that the springs can pull the

Name	Symbol	Range	Value(s)
**Nucleus radii** (unit: μm)			
Radius of soft core	R^S	5	5
Radius of hard core	R^{HS}	$[1.5, 3.5]$	1.5
Radius of hard core during M phase	R^{HM}	$[R^{HS}/R^S, 5]$	3.5
**Relative strength of forces** (unit: α)			
Stiffness of soft core of the nucleus	α^X	1	1
Stiffness of apical-nucleus spring	α^{aX}	$\mathcal{O}(1)$	2
Stiffness of nucleus-basal spring	α^{bX}	$\mathcal{O}(1)$	2
Stiffness of apical-apical spring	$\alpha^{a,S}$	$\mathcal{O}(10)$	5
Stiffness of apical-apical spring during G2 and M phases	$\alpha^{a,M}$	$> \alpha^{a,S}$	10
Magnitude of alignment force	α^{ab}	$\mathcal{O}(10)$	15
**Rate of actualization of the rest length of springs** (unit: $1/h$)			
Nucleus springs	k_η	≥ 2	5
Apical-apical springs	k_{a_0}	≥ 0	$\{0, 1\}$
**Basolateral adhesion**			
Rate of actualization of the basal points relative to the rate of actualization of apical points and nuclei during the minimization algorithm (basal adhesion)	ρ	$[0, 1]$	$\{0.5, 1\}$
Maximum distance between neighbouring basal points (lateral adhesion)	b_0	$\geq 1/6$	0.17
**Diffusion** (unit: $\mu m^2/h$)			
Diffusion coefficient	d	$[0, 25]$	$\{2.5, 62.5\}$
**Cell cycle** (unit: h)			
Minimum duration of cell cycle	$\sigma_{\min}$	10	$\{10, 10000\}$
Maximum duration of cell cycle	$\sigma_{\max}$	21	$\{21, 10000\}$
Duration of G2 phase	σ_{G2}	1/2	$\{0, 0.5\}$
Duration of M phase	σ_M	1/2	$\{0.1, 0.5\}$
**Daughter cells**			
Exclusion rate of one daughter cell per division	P_{out}	$[0, 100]\%$	$\{0, 60, 80, 100\}\%$

Table 1: Ranges of model parameters obtained from quantitative or qualitative experimental lab results (third column) and the values used in the paper (fourth column). The symbol $\mathcal{O}$ denotes order of magnitude. The space unit is μm (micrometer). The time unit is h (hour). The unit of strength of a force is α (strength of the soft core of the nucleus).

nucleus apicalwards. Therefore we have to choose $k_\eta \geq 2$ hours. Apical networks of epithelia are known to be contractile, however the contribution of contractility to the dynamics of pseudostratified epithelia is not yet understood. Therefore we do not have a criterion for choosing the rate of actualization of apical-apical springs k_{a_0}. Instead we studied two cases $k_{a_0} = 0$ and $k_{a_0} = 1$ corresponding to passive and contractile springs, respectively.

3.4 Basolateral adhesion

The parameter ρ should be chosen within $[0, 1]$, as explained in Section 2.1. Lateral adhesion is modelled by imposing a maximum distance between neighbouring basal points, which is

given by $2b_0\bar{R}$. If the tissue has a squared shape and 6 layers of nuclei, as observed in the 18-SS embryo in Figures 1C and 1D, then the distance between basal points should be approximately $2\bar{R}/6$. Therefore, we choose $b_0 \geq 1/6$.

3.5 Diffusion

The diffusion coefficient d has been estimated from the movement of nuclei in ex vivo movies from slice culture assays [3]. Given the successive positions of a nucleus over the life time of the cell, we selected a time window with 15 frames in which the nucleus is shaking without having any apparent directed movement. We measured the consecutive displacements Δx_n, $n = 1, ..., 14$ during this time window and obtained the mean $\hat{d}$ and standard deviation $\hat{\sigma}$ of the values $d_n = (\Delta x)^2_n/(2\Delta t)$, where $\Delta t = 0.1$ hours. We obtained $\hat{d} = 9.25\mu m^2/h$ and $\hat{\sigma} = 15.25\mu m^2/h$, so we consider the diffusion coefficient within $d \in [0, \hat{d}+\hat{\sigma}] \approx [0, 25]\mu m^2/h$.

3.6 Initial and stopping conditions

Name	Symbol	Value(s)/Range
Number of cells	N_0	30
	**Rest length of springs**	
Apical-nucleus springs	$\eta^{aX}_{i,0}$	1.5
Nucleus-basal springs	$\eta^{bX}_{i,0}$	1.5
Apical-apical springs	$a_{0,i,0}$	1/6
	**Cell cycle** (unit: h)	
Duration of cell cycle	$\sigma_{i,0}$	$\mathcal{U}([\sigma_{\min}, \sigma_{\max}])$
Birth time	$\tau_{i,0}$	$\mathcal{U}([-\sigma_{i,0}, 0])$
	**Cell positions** (unit: μm)	
Apical points	$a_{i,0}$	$[0, (N_0 - 1)5/3] \times \{50\}$
Basal points	$b_{i,0}$	$[0, (N_0 - 1)5/3] \times \{0\}$
Center of nuclei	$X_{i,0}$	$[0, (N_0 - 1)5/3] \times [0, 50]$
	**Stopping condition** (unit: h)	
Final time	T	48

Table 2: Initial and stopping conditions.

The initial number of cells is $N_0 = 30$. We define the positions of the apical $\mathbf{a}_0$ and basal $\mathbf{b}_0$ points along two parallel lines, respectively, at a distance $50\mu m$ from each other. In each line, the neighbouring points are placed at a distance $2\bar{R}/6 = 5/3\mu m$ from each other, giving to the tissue a squared shape $[0, (N_0 - 1)5/3] \times [0, 50]\mu m$ with space for 5 or 6 layers of nuclei in the apicobasal direction. Nuclei are then randomly thrown to the square and the minimization algorithm described in Section 2.1 is applied to the nuclei constrained to the square, while the apical and basal points are kept fixed. The result of the minimization algorithm gives $\mathbf{X}_0$. The rest lengths of the nucleus springs are initialized with relatively small values, namely $\eta^{aX}_{i,0} = \eta^{bX}_{i,0} = 1.5$, $i = 1, ..., N_0$, so that the apical network will be pulled down during the first time-iterations, which will force the nuclei and the springs to adjust to each other. The rest lengths of the apical-apical springs are initialized such that

the springs are initially at their rest-position, i.e., $2a_{0,i,0}\bar{R} = a_{i,0} - a_{i-1,0} = 2\bar{R}/6$, therefore, $a_{0,i,0} = 1/6,\ i = 1, ..., N-1$. For each cell $i = 1, ..., N$, we randomly generate the duration of cell cycle $\sigma_{i,0}$ from the uniform distribution $\mathcal{U}([\sigma_{\min}, \sigma_{\max}])$ and the birth time $\tau_{i,0}$ from $\mathcal{U}([-\sigma_{i,0}, 0])$. The final time is $T = 48$ hours.

4 Statistical quantifiers

In this Section we introduce the quantifiers associated to the evolution of tissue size and morphology that were used to obtain the graphs presented in the main text. The quantifiers are defined by:

- *Number of layers*, N_{layers}. We use a Voronoi tessellation [4] which divides the space in regions containing exactly one nucleus each and such that only the nuclei situated at the top layer of the tissue belong to a region with infinite area. We compute recursively each layer by successively identifying the regions with infinite area and removing them from the tessellation.

- *Width of apical, nuclear and basal domains (unit: μm)*,

  $$W_{\text{apical}} = a_N(1) - a_1(1), \quad W_{\text{nuclei}} = x_{\text{right}} - x_{\text{left}} \quad \text{and} \quad W_{\text{basal}} = b_N(1) - b_1(1),$$

  where $x_{\text{left}} = \min_i X_i(1)$, $x_{\text{right}} = \max_i X_i(1)$.

- *Apicobasal length and nuclear-basal length (unit: μm)*,

  $$L_{AB} = \frac{1}{\#S_{\text{middle}}} \sum_{i \in S_{\text{middle}}} |a_i(2) - b_i(2)|$$

  and

  $$L_{NB} = \frac{1}{\#S_{\text{middle}}} \sum_{i \in S_{\text{middle}}} |X_i(2) - b_i(2)|,$$

  where $S_{\text{middle}} = \{i : x_{\text{left}} + W_{\text{nuclei}}/4 \leq X_i(1) < x_{\text{right}} - W_{\text{nuclei}}/4\}$ and $\#S_{\text{middle}}$ is the number of elements in S_{middle}.

- *Position of mitosis of cell i*

  $$P_{\text{div}} = \frac{|X_i^n(2) - b_i^n(2)|}{|a_i^n(2) - b_i^n(2)|}, \text{ with } t^n < \tau_i + \sigma_i < t^{n+1}.$$

- *Straightness of apical network*,

  $$A_{\text{straight}} = \left(\frac{1}{|a_1 - a_N|} \sum_{i=1}^{N-1} |a_i - a_{i+1}| \right)^{-1}$$

We run the simulation of the tissue evolution for p different initial conditions, with $p = 10$. For each time $t_n = n\Delta t$, we compute the average and standard deviation of each quantifier over the results of the p simulations. The statistics of P_{div} are computed over the whole dynamics. The results are plotted in Figures 3, 4, 5 and 7 and in Supplementary Figures 5 and 6 for the parameters presented in Table 1 and Supplementary Table 1 and initial conditions presented in Table 2.

References

[1] Degond P, Ferreira MA, Motsch S. Damped Arrow-Hurwicz algorithm for sphere packing. *Journal of Computational Physics*. 2017; 332:47-65.

[2] Peterson, DW. A review of constraint qualifications in finite-dimensional spaces. *SIAM Review*. 1973; 15(3):639-654.

[3] Das RM, Wilcock AC, Swedlow JR, Storey KG. High-resolution live imaging of cell behavior in the developing neuroepithelium. *Journal of Visualized Experiments: JoVE*. 2012; 62.

[4] Debnath D, Gainer JS, Kilic C, Kim D, Matchev KT, Yang YP. Identifying phase-space boundaries with Voronoi tessellations. *European Physical Journal C*. 2016; 76(11), 645.
