# Supplementary material for: Interkinetic nuclear movements promote apical expansion in pseudostratified epithelia at the expense of apicobasal elongation
Source: PLoS Comput Biol. 2019 Dec 23;15(12):e1007171. doi: 10.1371/journal.pcbi.1007171 (PMC6957215; doi:10.1371/journal.pcbi.1007171)
Supplement: Attachment — Submitted filename: 2019 Ferreira et al Plos CB Answers to reviewers.docx [file pcbi.1007171.s016.docx]

**Point-by-point answers to reviewers’ comments**

First of all, we would like to thank the editor and reviewers for taking the time to assess the relevance and quality of our work. We were pleased to see that the reviewers deemed the work to be timely and the data of potential immediate and broad interest. Please find below detailed point-by-point answers to all the reviewers comments.

**Comments to the Authors:**

Reviewer #1: Studies of interkinetic nuclear migration are now *en vogue* in developmental biology, partly due to its association with cell differentiation and congenital defects. This timely manuscript by Ferreira and colleagues combines theoretical and experimental approaches to provide novel insight into this fundamental cell behavior which could not have been achieved with either method alone. This manuscript is therefore expected to be of immediate interest to a broad field. A few limitations should be addressed:

1) Where are the Rock-inhibition methods detailed? The figure legend states explants were cultured in suspension in the presence of 400 µM Y27632. This is a very high concentration (manufacturer maximum solubility <200 mM in DMSO so would need >0.2% DMSO in culture) under sub-optimal culture conditions. It could cause cell death and delamination, which would explain changes observed in Supp Fig 2. Explant viability should be confirmed.

**We are sorry to have omitted the paragraph corresponding to the drug treatment. It has now been added to the Materials and Methods section. Regarding the concentration of the ROCK inhibitor guidelines for working concentrations for cell monolayers in 2D-culture, where the drug will be in immediate contact with cells, cannot be applied to whole explants. In whole explant culture, the drug needs to pass the ectoderm, the mesoderm (dermomyotome and sclerotome) before reaching the neural tube. Importantly, we do not analyze sections located at the edge of the explants because these regions undergo wound healing and thus, we are only looking at the neural tube located deep inside the explanted region. In addition, to avoid possible effect on cell cycle/proliferation, we performed short treatments (2 hours only). Therefore, higher concentrations need to be used for a sufficient amount of drug to reach the region of interest within the time scale of the experiment. Importantly, the DAPI staining indicates that nuclei were OK as no fragmented nuclei were observed in controls, DMSO or ROCK inhibitor treated explants.**

**To further assess tissue viability, we have analyzed cell death by TUNEL assay (protocol added to the Methods) and found no induction of cell death after exposure to 400µM of ROCK inhibitor and have added these data to new supplementary figure S3.**

2a) The phrase “apical constriction” appears to be used loosely to mean the average tension across the apical surface, rather than the actomyosin-dependent pulsatile contractility of individual cells as biologists will understand it. This needs to be clarified.

**Thank you for this suggestion. We have now replaced “apical constriction” by “apical contractility” throughout the manuscript which is more accurate description.**

2b) Rather than defining each cell’s apical surface as a single point, it may be more meaningful to describe it as the non-zero space between adjacent apical points (i.e. the springs, with the points representing cell-cell adhesions instead). Given the apical areas of cells change predictably during IKNM (which could be confirmed in the model used here), it may be meaningful to relate individual cell preferred apical lengths to IKNM phases. Alternatively, each cell could be related to an adjacent apical spring as its apical surface and contractility of individual springs could be varied.

**This is an interesting comment. There are indeed various alternatives to model the apical surface each with their own merit. However, we think that modelling the apical surface as an interval rather than a point would allow for more details in the model but at the expense of extra modelling parameters which would need to be tuned. Here we have focused on nuclei packing which is why we have provided a coarse description of the cytoplasm and apical and basal poles. In a future work, the role of the cytoplasm and apical/basal membrane could be further investigated but this should be done systematically. Given the current version of the model we deemed inappropriate to provide a detailed description of the apical side while still providing a coarse description of the rest of the cell.**

3) The expansion and eversion of the apical surface in simulations under conditions of passive apical springs and low extrusion is very intriguing. It is reminiscent of the morphology of the neuroepithelium in the open neuropores of Rock-inhibited embryos (e.g. Escuin et al, J Cell Sci, 2015). However, it is difficult to picture the relevance of this eversion to the closed neural tube. The authors should establish whether imposing boundary conditions which limit dorso-ventral expansion, as is the case in vivo, compensates for the role of contractile apical springs (Figure 5).

**As suggested, we repeated simulations from Figure 5 with passive apical springs but imposed lateral walls to restrict dorso-ventral expansion. The results show that imposing lateral boundaries is not sufficient to compensate for the lack of apical contractility. These data were added as new supplementary Figure S5.**

**The closed neural tube in the trunk region, despite its name is not shaped as an actual tube from a geometrical point of view. It is formed of two flat epithelial arrangements linked on their dorsal and ventral sides by two regions undergoing extensive bending in which cells are much shorter along the apicobasal axis: the roof plate and the floor plate. Thus, the epithelial organization displayed in the simulations is relevant to the actual organization of the intermediate portion of the pseudostratified trunk neuroepithelium.**

4a) The authors interpret the change in shape of the nuclei as indicating that “nuclei are slightly compressed along the dorsoventral axis, giving them an elongated form along the apicobasal axis”. The interpretation of this shape change is not unequivocal. For example, apical pulling of the nucleus during PRAM through an increasingly apically-crowded epithelium could favour a more elongated shape. Experimental verification of tension anisotropy (e.g. with tissue cut and deform experiments) should be provided.

**We agree that apical pulling could contribute to nuclear elongation. However, at any given time point the proportion of nuclei being apically pulled is too low to explain that nearly 90% of all nuclei are elongated. Further, nuclear shape becomes more elongated between 2 days and 3 days of development and then nuclear shape stabilizes. Average cell cycle durations between early and late stages of neural tube development progressively increase. That means that the relative importance of PRAM over time decreases (representing a smaller portion of the total cell cycle in late stages than early ones). If apical pulling during PRAM was a major contributor of nuclear shape, nuclei should get less elongated as development progresses due to the reduction of the relative importance of PRAM over time which is not the case. Furthermore, nuclei are rounder in regions of lower density strongly suggesting that local packing is a driving force of nuclear shape. Altogether, these observations argue against a major role of apical pulling in setting the mean nuclear shape.**

**We are not sure how this reviewer wanted us to perform a “cut and deform” experiment. The experiments performed with inhibitors were done on explants encompassing a 10-somite long trunk region containing all tissues (surface ectoderm, somites, lateral plate, endoderm, neural tube and notochord). Cutting such explants does not lead to any noticeable changes in nucleus, cell or tissue shape. From the comment, we interpreted that the question was whether relaxing constraints across the neural tube would lead to less elongated nuclei. Cells are attached to one another and anchored to the substrate. Thus, relaxing constraints means impairing cell-cell or cell-matrix adhesion. Inhibiting actomyosin contractility (using the ROCK inhibitor) affect cell-cell adhesion since contractility and cell-cell adhesion are coupled via the actin network. The ROCK inhibitor treatment indeed led to rounder nuclei.**

**To affect cell-matrix adhesion, we treated trunk explants with Dispase to degrade collagens and Fibronectin, thus reducing basal attachment. This treatment led to a shortening of the apicobasal length and rounding of nuclei. These data were added to supplementary figure 4. These data further support the idea that relaxing the neuroepithelium either by targeting apical contractility (ROCK inhibitor) or basal attachment (Dispase) leads to less elongated cell and nuclear shapes.**

4b) Can the authors comment on whether apical crowding alters PRAM rate in their simulation, for example due to increased drag?

**We are not sure what PRAM rates actually means here. In the model, the duration of PRAM is set by the internal clock of the cell and is equal for all cells. In other words, all cells pull their nuclei apically for the same amount of time. Therefore, if the PRAM rate is the ratio between the duration of PRAM and the total length of the cell cycle, this ratio is constant across simulations regardless of crowding. However, the reference to the putative effect of nuclear crowding on drag made us think that the reviewer wanted to know whether nuclear crowding would affect the speed at which nuclei travel towards the apical side of the tissue. We ran 12-hour simulations with an exclusion rate of 50% to keep cell number constant and assessed the effect of changing the diameter of the hard core of the nucleus outside division (Rh) and during division (Rh_div). In all previous simulation Rh/Rh_div were respectively set to 0.3 and 0.7 of the soft core diameter. We tried various combinations (see below). We also affected crowding by increasing the strength of the repulsion force between soft cores of nuclei (alpha X) increasing from 1 to 2 with Rh /Rh_div set to 0.3 and 0.7.**

**We plotted the nuclear packing fraction, volume occupied by the nuclei hard-cores divided by the volume of the tissue (V{nuclei} / V{tissue}), in the apical half of the tissue (panel A of the figure below) in parallel with the mean apicobasal nuclear velocity during activeG2/M (panel B of the figure below). Indeed, lowering packing increases mean nuclear speed during PRAM while increasing packing reduces it.**

**Impact of modulating apical nuclear packing on the mean apicobasal velocity of nuclei during PRAM (active G2/M).**

A. Packing nuclear fraction in the apical half of the PSE (V{nuclei / V{tissue}). B. Mean apicobasal nuclear velocity during PRAM (active G2/M) in micron/hour. White boxes represent conditions used throughout the paper (Rh0.3/Rh_div0.7). Grey boxes depict variations in hard core diameter during interphase (Rh) and division (Rh_div). Grey boxes with a brown outline represent the condition with normal Rh/Rh_div coupled with a doubling of the repulsion force between the soft cores of nuclei.

**Interestingly, large increase (+23%) or reduction (-19, 65, 86%) of packing via changes in hard core diameters (Rh/Rh_div) only translate in moderate changes of mean velocity during PRAM (respectively, -3%, +4, 5.5, and 8.5%) whereas a reduction of 12% of nuclear packing by doubling the repulsion between soft cores led to an increase of 11% in terms of mean nuclear velocity. Overall, these simulations indicate that indeed mean apicobasal nuclear velocity during PRAM responds to apical nuclear crowding. Since these data are slightly out of the scope of the manuscript and that we were just asked to comment on that point, we have not added these data in the revised version of the manuscript.**

5) Modelling a straight portion of PSE is very strategic, circumventing the emerging differences between IKNM in tissues with different geometries. In vivo most PSEs are curved. It would be informative to model or at least discuss the effect of basal curvature (e.g. as in the retina).

**PSE come in various shapes and sizes and often include a mix of straight portions as in the neural tube where the intermediate region is straight while the roof plate and floor plates are curved. A curved portion can be roughly approximated by modelling cells in which the basal side is larger than the apical side. We have discussed this point using the floor plate as an example. Regarding this comment, we have added a comment about the putative interplay between curvature and cell cycle phases or actomyosin contractility in the discussion. However, our model does not currently allow us to test for the effect of curvature and we think that it is beyond the scope of this study.**

6) Cell adhesion is repeatedly mentioned throughout the manuscript. The model only incorporates the apical-most cell adhesions (i.e. tight junctions). In reality, other junctions such as N-cadherin adherens junctions extend laterally. Their likely impact on elongation and the requirement for active apical tension should be discussed.

**Cell-cell adhesion is actually included in the model in multiple ways. As mentioned, there is the link between adjacent apical points. In addition, basal points are not totally free to move. As described in the supplementary information and in the main text, we impose a maximum distance between adjacent basal points thus restricting how much cells can flatten onto the basal substrate. In cells, basolateral adhesion opposes flattening of cells onto the substrate and therefore, imposing a maximal distance between adjacent basal points in the model corresponds, at least partially, to lateral adhesion between cells.**

Minor comments:
7) The authors may wish to cite the observation in Xenopus embryos that their neuroepithelium elongates apicobasally during closure despite not being pseudostratified (e.g. see Inoue et al, Biomech Model Mechanobiol,2016). This supports their conclusion that the two processes are likely to be independent.

**Thank you for this suggestion we have now added this reference in the discussion**

8) CAKUT should be defined where it is first used in the introduction, not in the discussion.

**Thank you. We now describe CAKUT in full when first used. Given that CAKUT is not a very well-known acronym and is only used twice in the manuscript we decided to leave it alongside its definition in the discussion as well.**

9) The representative images shown in Supp Fig 2 suggest the dorsoventral length (i.e. roof plate to floor plate) of Rock-inhibited embryos is longer than controls. Is this reproducible? What might this mean (given 2 hours is likely to be too short for it to be the consequence of changes in IKNM)?

**Yes, it is reproducible. Given the short time of the experiment, it is fair to assume that cell volume is constant. Thus, it means that, due to apical relaxation, cells get shorter along the apicobasal axis while getting larger in the perpendicular axes (DV, AP). A switch akin to a rectangular-to-square shape change. If each cell undergoes such change the whole tissue changes shape. Similar changes were modeled before as a result of the balance between basal adhesion and apical tension (e.g. Hannezo et al PNAS 2014) and in extreme cases correspond to a transition from columnar to squamous epithelia.**

**We observe similar changes when treating trunk explants with dispase. That also is reproducible. In fact, dispase is used to dissect neural tubes for cell cultures and one knows that neural tubes are ready to dissect when they start to buckle and their extremities stick out from underneath the ectoderm indicating that they elongated significantly along their AP axis as a consequence of detachment from the matrix. We have shown that detaching the neuroepithelium from its basal extracellular matrix reduces the apicobasal length suggesting a similar change in cell shape from rectangular to square leading to a change in tissue shape (reduction of the AB length together with an increase of AP and DV length).**

10) The authors discuss various potential explanations for why apical crowding only occurs after the neural tube closes. The apical surface of the open posterior neuropore is longer than the length of the closed lumen. Again, the change in boundary conditions between the open and closed neural tube may affect apical crowding, stratification and elongation.

**That is an interesting point. However, we have not discussed the emergence of apical crowding as being temporally linked with the actual time of neural tube closure. Apical nuclear crowding is only detected several hours after the tube has completed closure. The two events are unlikely to be causally linked. The first physiological time point we mention in our data is at two days of incubation. Apical nuclear crowding is not yet in place whereas dorsal neural tube closure is already completed.**

------------------------------------
Reviewer #2: The manuscript by Ferreira and colleagues examines the potential roles of interkinetic movements of nuclei (INM) for the growth and shape of pseudostratified epithelia using the developing chick neural tube as a model. They use 2D computational modelling and simulations together with embryo observations and some limited experimental manipulation. As the neural tube grows, they observe changes in nuclear shape, and cell size and shape and number of nuclei and of pseudostratified layers. The model examines how these localized changes could result in tissue level changes. Overall, they propose that INM is important for the expansion of the apical domain of the epithelium. Furthermore, their data suggests that the apicobasal elongation of neuroepithelial cells is not an emergent property but instead requires a separate elongation program.

The authors explain in detail the assumptions made for the mathematical modelling and the parameters used for the simulations. They make some insightful observations which may be of more general importance and may also be relevant to other pseudostratified epithelia. The data are well-presented and discussed.

The experimental manipulations are limited to exposure of neuroepithelium to ROCK inhibitor and the authors may consider using inhibitors of additional cellular processes.

**We have now included the effect of reducing basal attachment by treating the explants with dispase. These data show that impair attachment via partial extracellular matrix degradation leads to a change of nuclear, cell and tissue shape (supp figure S4). We also added the inhibition of Rac1 activity for comparison with that of actomyosin contractility (data added to supp figure S2)**

-----------------------------------------------------
Reviewer #3: In this paper, an original 2D off-lattice agent-based model for the dynamics of pseudostratified epithelia is presented.

Computational simulations of the model are combined with experimental results on the growth of the chick neuroepithelium to assess the impact of interkinetic movement versus other cytoskeleton-dependent processes, such as adhesion and mitosis, on the dynamics of pseudostratified epithelia.

In the mathematical model, each cell is approximated as a nucleus, an apical point and a basal point. The cell nucleus is represented as an inner hard sphere surrounded by an outer soft sphere. The deformation occurring when cell nuclei are pressed against each other is incorporated into the model by allowing the soft spheres to overlap, while the consequent repulsion between the nuclei is modelled by imposing a dynamical non-overlapping constraint between the hard spheres. The cell cytoplasm is seen as a viscoelastic material modelled through as a set of springs linking the nuclei to the apical and basal points.

Cell division and both cell-cell and cell-matrix interactions are incorporated into the agent-based model through a set of mechanical and behavioral rules, which are elegantly integrated with an energy minimization algorithm ensuring that the non-overlapping constraint between the hard parts of the cell nuclei is satisfied.

The paper is pleasant to read, the presentation is clear and the results are interesting. The model appears to be original, based on sound modelling strategies and of potential interest to applied mathematicians and physicists working on the mathematical modelling of tissue mechanics. The short summary of the model presented in the paper is effective and nicely written. Moreover, a clear and detailed description of the model is provided in the Supplementary Information along with a precise account of the model parameterisation and the details of computational simulations. As a mathematician, I cannot comment on the quality of the experimental results but the conclusions drawn from the outcomes of the model appear to be coherent and interesting.

**Have all data underlying the figures and results presented in the manuscript been provided?**
Large-scale datasets should be made available via a public repository as described in the *PLOS Computational Biology* [data availability policy](http://journals.plos.org/ploscompbiol/s/data-availability), and numerical data that underlies graphs or summary statistics should be provided in spreadsheet form as supporting information.

Reviewer #1: No: Data that underlies graphs not provided in spreadsheet form as supporting information

**We have now included a Supplementary Spreadsheet with all the raw data.**

Reviewer #2: Yes

Reviewer #3: Yes
